# Estimating the fiscal impact of rare diseases using a public economic framework: a case study applied to hereditary transthyretin-mediated (hATTR) amyloidosis

**DOI:** 10.1186/s13023-019-1199-x

**Published:** 2019-09-18

**Authors:** Mark P. Connolly, Saswat Panda, Julien Patris, Bouke P. C. Hazenberg

**Affiliations:** 1 Global Market Access Solutions Sarl, St-Prex, Switzerland; 2 Unit of Pharmacoepidemiology & Pharmacoeconomics, Department of Pharmacy, University of Groningen, Groningen, The Netherlands; 3 Alnylam Pharmaceuticals, Strawinskylaan 3051, 1077 ZX Amsterdam, The Netherlands; 4 Department of Rheumatology & Clinical Immunology, University of Groningen, University Medical Center Groningen, Groningen, The Netherlands

**Keywords:** Public economics, Generational accounting, Rare diseases, Fiscal analysis, Hereditary transthyretin-derived (hATTR) amyloidosis

## Abstract

**Background:**

A wide range of rare diseases can have fiscal impacts on government finances that extend beyond expected healthcare costs. Conditions preventing people from achieving national lifetime work averages will influence lifetime taxes paid and increase the likelihood of dependence on public income support. Consequently, interventions that influence projected lifetime work activity, morbidity and mortality can have positive and negative fiscal consequences for government. The aim of this study was to apply a public economic framework to a rare disease that takes into consideration a broad range of costs that are relevant to government in relation to transfers received and taxes paid. As a case study we constructed a simulation model to calculate the fiscal life course of an individual with hereditary transthyretin-mediated (hATTR) amyloidosis in The Netherlands. In this lethal disease different progressive disease scenarios occur, including polyneuropathy and/or cardiomyopathy.

**Results:**

Due to progressive disability, health care resource use, and early death, hATTR amyloidosis with polyneuropathy receives more transfers from government compared to the general population. In a scenario where a patient is diagnoses with hATTR at age 45, an individual pays €180,812 less in lifetime taxes and receives incrementally €111,695 in transfers from the government, compared to a person without hATTR. Patients suffering from cardiomyopathy die after median 4 years. The health costs of this scenario are therefore lower than that of the other polyneuropathy-based scenarios.

**Conclusions:**

The fiscal analysis illustrates how health conditions influence not only health costs, but also the cross-sectorial public economic burden attributed to lost tax revenues and public disability allowances. Due to the progressive nature of hATTR amyloidosis used in this study, public costs including disability increase as the disease progresses with reduced lifetime taxes paid. The results indicate that halting disease progression early in the disease course would generate fiscal benefits beyond health benefits for patients. This analysis highlights the fiscal consequences of diseases and the need for broader perspectives applied to evaluate health conditions. Conventional cost-effectiveness framework used by many health technology assessment agencies have well-documented limitations in the field of rare diseases and fiscal modeling should be a complementary approach to consider.

## Background

The burden of health conditions is often measured from the healthcare perspective in terms of direct health costs attributed to a medical condition at specific stages of the disease and attributed health costs that stop at death or remission. There is a broader perspective that can be applied to investments in healthcare interventions using human capital economics that is seldom considered. This perspective can reflect the impact of health on the government finances based on lifetime taxes paid and demand for public benefits linked to disability status attributed to health status [1–3]. The government perspective is particularly relevant in tax finance health systems where public money is used to pay for healthcare which is the dominant funder in OECD countries [4]. In contrast with the healthcare perspective, the government public economic perspective can have consequences even in death as health events and disability represent unfulfilled lifetime taxes paid, increased social dependency costs and pension receipts. This perspective implicates that budget impact analysis could be broadened to include the impact of health and technologies across all government budgets, not just health, and how changes in health status will influence other government budgets [1].

Several frameworks are available for capturing the financial relationship between citizens and state and the lifetime transactions that occur. Examples include generational accounting models, microsimulation models (STINMOD) and social accounting matrix (SAM) often used by central governments for evaluating the impact of policy decisions on current and future generations [5–9]. Current government promises in the form of income support, disability support, pensions and healthcare are often included for evaluating cross-sector impact of policies. Similarly, lifetime tax revenue projections are included to estimate how population dynamics and workforce participation will influence government revenue. Underlying these modeling frameworks are age-specific cohorts and wage income which determines government future revenue from direct and indirect taxes. Variations in population health norms due to morbidity or early mortality can be used to project the cross-sectorial impact of health on government. Applying such a framework makes it possible to evaluate how changes in public health and investments in healthcare interventions influence other government budgets beyond health [1, 10].

Public economic frameworks applied to health conditions often illustrate the cross-sectorial impact of health on other government sectors including disability payments, early retirement, living allowances for maintaining living standards, and lost tax revenues [11]. The magnitude of the public economic impact is linked to both the severity of the condition, the age at which health deteriorates, and the likelihood to remain working or partially employed.

The aim of this study was to explore the applicability of applying a broader public economic perspective to evaluate a rare disease called hereditary transthyretin-mediated (hATTR) amyloidosis.

### hATTR amyloidosis

Amyloidosis is a rare and multiorgan disease that results in progressive, chronically debilitating morbidity and increased mortality. hATTR amyloidosis is caused by a genetic mutation in the transthyretin (*TTR*) gene that leads to misfolding of TTR proteins, aggregation of these TTR proteins into amyloid fibrils, and accumulation of these amyloid fibrils in multiple tissues and organs throughout the body, affecting the nerves, the heart, eyes, and the gastrointestinal tract [12–17]. The clinical presentation of hATTR amyloidosis includes sensory, motor, and autonomic neuropathy as well as cardiomyopathy. Polyneuropathy manifests as peripheral neuropathy, autonomic dysfunction, and/or motor weakness in many cases, making it increasingly difficult for many patients to perform physical activity and work [18]. Studies have demonstrated that of the 33.3% patients that are employed, 21.9% report missing work time due to the disease, and 40.7% report some impairment at work from the disease [19]. Cardiomyopathy is also a serious disease manifestation; in an observational study of 77 patients in The Netherlands, cardiac involvement was present in half of the patients presenting with hATTR amyloidosis, regardless of initial clinical presentation and genotype [20]. hATTR amyloidosis can lead to significant morbidity and disability, with a median survival of 10–12 years following diagnosis and a strongly reduced survival of 4 years in those patients presenting with cardiomyopathy [20–22].

Scoring systems are available for assessing hATTR amyloidosis disease stage with the polyneuropathy disability (PND) score being commonly used [13]. Previous studies reported the relation between PND scores and the likelihood of work [18]; hence this is a useful relationship for estimating the fiscal consequences of morbidity from the perspective of government.

In the context of the fiscal model we seek to understand how hATTR amyloidosis morbidity, linked to employment activity based on PND score, and mortality will influence government costs in The Netherlands. We believe this framework examines the broader societal impact of rare diseases, as well as the potential societal benefits of addressing them. Current Value Assessment tools typically ignore these benefits, and we therefore believe that alternative value assessment framework such as fiscal modelling should be more routinely used or accepted to inform the broader value of technologies.

## Methods

### Model design

A fiscal cost model was developed for estimating public economic consequences of hATTR amyloidosis in the Netherlands. The framework was built based on similar frameworks used to evaluate investments in healthcare technologies and to estimate disease burden [3, 23]. The calculator estimates the difference between the average population adjusted for age-specific mortality and disability and simulated individual scenarios of hATTR amyloidosis patient journeys in order to estimate the incremental public economic impact. The analysis estimates lifetime direct and indirect taxes paid in both cohorts and the incremental benefits received in terms of disability payments, old-age pensions, and healthcare resource use costs. Taxes consist of direct taxes on earnings, and indirect taxes which are calculated by using a flat VAT tax rate on taxable disposable income.

The analysis first required constructing the average fiscal life course for the general population adjusted for age-specific mortality. We apply age-specific income adjusted for labor force participation rate to derive direct taxes paid to government [24, 25]. Standard published direct tax rates for the Netherlands were applied to age-specific income data [26]. We then applied disposable income to estimate the age-specific indirect taxes paid to VAT through consumption (the VAT tax rate used was 21%) [27, 28].

Similarly, we apply rate of retirement to the general population to indicate transition from employment to pension which changes the annual income and taxes paid. The retirement age, productivity growth, discount rate for costs, and cost-inflation rate are set to 65, 1, 4, and 1.5% for our analysis. Productivity growth was estimated to be 1% by calculating the geometric mean of productivity growth between 1990 to 2017 for the Netherlands [29]. Cost inflation was estimated to be 1.5% by figuring the geometric mean of past inflation trends in the Netherlands from 2001 to 2018 [30]. We used a retirement age of 65 based on the current Dutch policy on retirement [31]. We use a 4% discount rate per Dutch Health Economic Guidelines [32].

### Health care costs

Healthcare costs by PND score were derived from a resource use survey in the Netherlands and applying local unit costs to each item. The health cost survey was based on best supportive care excluding liver transplantation and TTR tetramer-stabilizing treatment in which we applied estimated resource use patterns by PND score to known cost tariffs. The healthcare costs for those in the non-hATTR amyloidosis population were derived from the PAID (Practical Application to Include future Disease costs) calculator, version 1.1, a tool to estimate per capita indirect medical costs for the Netherlands [33]. The diseases which resemble symptoms of hATTR amyloidosis were deselected, and the results of the calculator were used for determining per capita health care expenditure in the non-hATTR amyloidosis population [34]. The output from the health cost analysis are provided in Table 1, and health resource unit costs are provided in the Additional file 1.

**Table 1:** Health care resource use hATTR amyloidosis best supportive care by NT proBNP^a^ status

Best Supportive Care (BSC)	HCRU PN^b^ (1-year cost)	HCRU CM^c^ (1-year cost)
NT proBNP< 3000 pg/mL	221	18,375
	4077	18,375
	6295	18,375
	11,455	18,375
	16,397	18,375
	167,247	18,375
NT proBNP≥3000 pg/mL	221	25,982
	4077	25,982
	6295	25,982
	11,455	25,982
	16,397	25,982
	167,247	25,982

^a^N-terminal pro b-type natriuretic peptide; ^b^: Healthcare resource use, polyneuropathy; ^c^: Healthcare resource use, severe cardiomyopathy

### Economic inputs

Age-specific earnings, labor force participation, unemployment, old age pension, and disability pension figures were obtained from the Statistics Netherlands’ data portal [35].

### Disability payments

Disability payments in the Netherlands are linked to disability level determined by medical experts with fixed percentages of disability payments available based on earnings and projected wage losses. To account for hATTR amyloidosis disability, we map disability scores to different percentage disability defined by Dutch law [36].

### Patient scenarios

In hATTR amyloidosis, disease progression and mortality vary widely, in particular when cardiac involvement is present [18]. Variation in disease progression and mortality are largely dependent on the type of mutation of a particular patient. Consequently, it is difficult to model disease progression that reflects a typical cohort. To overcome this challenge, we have defined typical scenarios from published studies [18, 37, 38].

We assume that after someone has been diagnosed with hATTR amyloidosis, they will have some form of polyneuropathy disability (PND) over time, which worsens until death from PND, other co-morbidities, or other causes. We assume that there are no back transitions from more severe PND stages to less severe PND stages. We assume PND progression is non-reversible.

We opted to use a scenario-based approach to derive results, as patients will have different experiences with hATTR amyloidosis. As such, we developed scenarios for how long a patient may stay in each PND score based on real world evidence on ranges of time for disease duration, presented in the published literature [37, 38]. The median value for disease duration was used to develop a scenario where a patient might have a “median progression” through disease states—transitioning to severe disease as expected. The maximum value for disease duration was used to present the extreme case where a patient may live with hATTR amyloidosis for the maximum observed time. We also designed scenarios to have an early disease onset and late disease onset, where the patient is diagnosed at age 45 (scenarios 1 and 2) and age 60 (scenarios 3 and 4), respectively. These values were used as they are plausible ages where many patients are diagnosed in the literature [20, 37, 38]. Finally, we designed a scenario to highlight the impact of cardiomyopathy as main manifestation of hATTR amyloidosis. This scenario is meant to emphasize the severity of having cardiomyopathy in a patient with hATTR amyloidosis. This scenario accounts for the fact that the median survival of severe cardiomyopathy is about 4 years, after which the patient dies as a result of complications caused by cardiomyopathy, rather than through progression to the last stage of polyneuropathy [39]. The scenarios were also evaluated with a local expert to ensure that these are plausible disease profiles of patients (Table 2).

**Table 2:** Description of simulated disease scenarios assessed fiscally

Scenario 1	Early disease onset, at age 45 at PND state 1, with a median progression through PND states, and death at age 55 at PND state 4, without severe cardiomyopathy.
Scenario 2	Early disease onset, at age 45 at PND state 1, with a slow progression through PND states, and death at age 65 at PND state 4, without severe cardiomyopathy.
Scenario 3	Late disease onset, at age 60 at PND state 1, with a median progression through PND states, and death at age 70 at PND state 4, without severe cardiomyopathy.
Scenario 4	Late disease onset and late diagnosis, at age 60 during PND state 2, in a patient with severe cardiomyopathy, where the patient dies within 4 years, at PND state 3b, before reaching PND state 4.

## Results

**Fig. 1:**
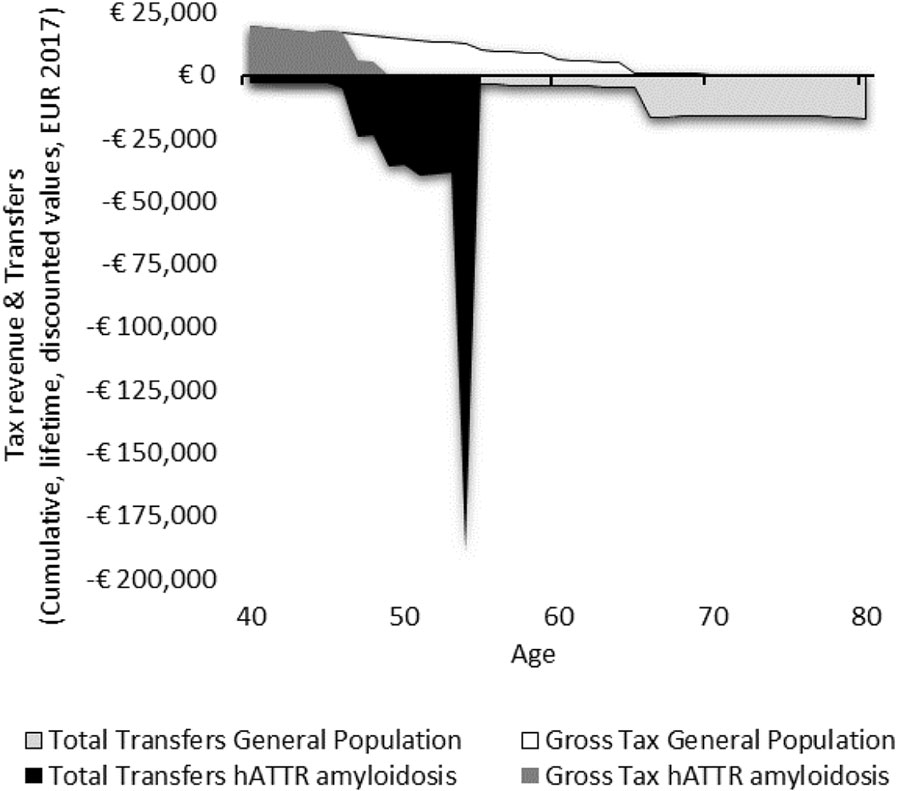
Lifetime transfers and taxes for Scenario 1

**Fig. 2:**
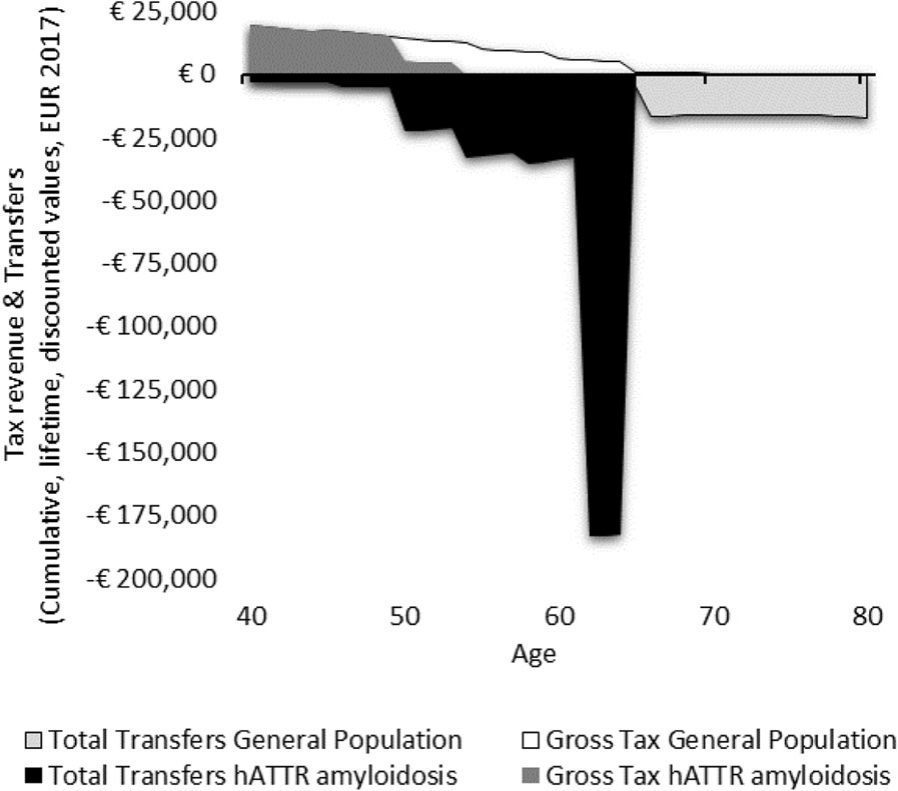
Lifetime transfers and taxes for Scenario 2

**Fig. 3:**
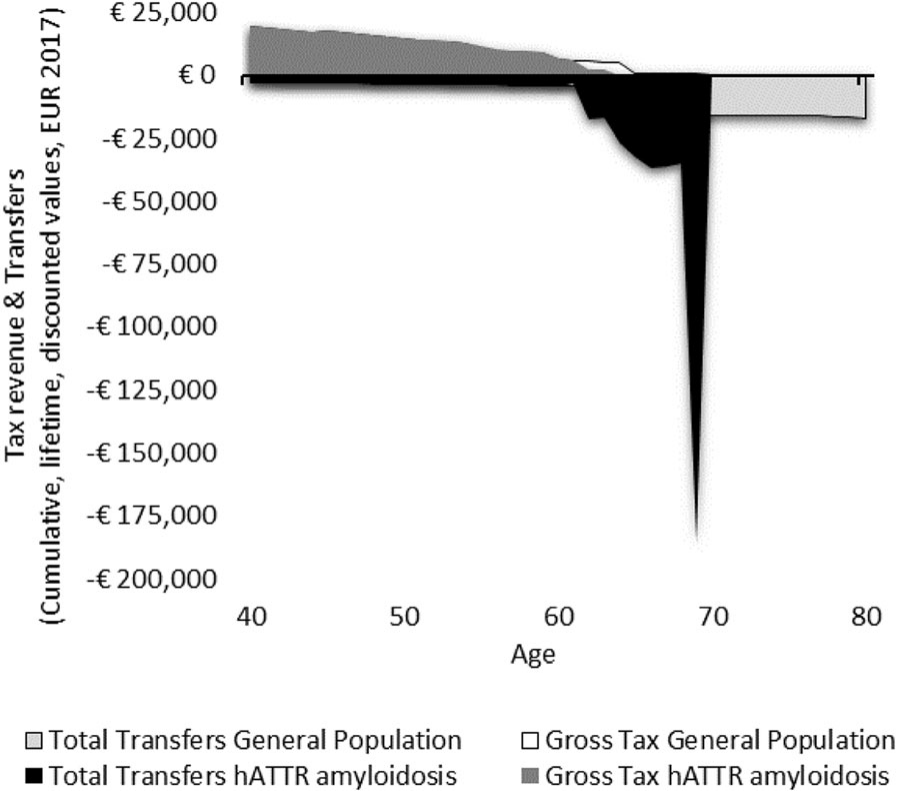
Lifetime transfers and taxes for Scenario 3

We estimate that in the general population between the timeframe of ages 40 and 80, a person receives €338,330, and pays €319,922 in taxes (Table 3). Scenarios 1–3 illustrate hypothetical cases where a patient has hATTR amyloidosis without severe cardiomyopathy. In Scenario 1 (Fig. 1), where a patient has an early disease onset with a median disease progression, resulting in early death at age 55—their lifetime earnings are diminished, and lifetime taxes are diminished. In scenario 1, the person would pay €180,812 less in taxes. Conversely, transfers are far greater than that of the general population, in this scenario, where a person receives €111,695 more in pensions, disability payments, and healthcare from the government. Although Scenario 2 (Fig. 2) is a case where the patient lives ten additional years with the disease, their earning potential is only somewhat greater than Scenario 1, as much of the later years of life are years lived in disability. The lifetime earnings of a person in Scenario 2 are €348,952, while the earnings of a person in Scenario 1 are €247,559. Scenario 3 (Fig. 3) shows the profile of a person with a late disease onset and median disease progression, and this scenario accounts for the impact of receiving old age pensions from the government for the time alive after retirement.

**Table 3:** The estimated per person economic impact between the ages 40–80 attributed to patients with hATTR amyloidosis in different disease presentation scenarios

Fiscal Parameters	General Population (Comparator)	Scenario 1		Scenario 2		Scenario 3		Scenario 4	
		hATTR Amyloidosis Population	Difference	hATTR Amyloidosis Population	Difference	hATTR Amyloidosis Population	Difference	hATTR Amyloidosis Population	Difference
Transfer Payments									
Government transfers	€ 2150	€ 180,949	(€ 178,799)	€ 285,355	(€ 283,204)	€ 84,011	(€ 81,861)	€ 55,369	(€ 53,219)
Pensions	€ 130,082	€ 0	€ 130,082	€ 0	€ 130,082	€ 55,527	€ 74,556	€ 0	€ 130,082
Health costs	€ 206,098	€ 269,076	(€ 62,979)	€ 656,129	(€ 450,031)	€ 322,218	(€ 116,120)	€ 110,502	€ 95,596
Total transfers	€ 338,330	€ 450,025	(€ 111,695)	€ 941,483	(€ 603,153)	€ 461,756	(€ 123,426)	€ 165,871	€ 172,459
Societal									
Lifetime Earnings	€ 577,114	€ 247,559	€ 329,555	€ 348,952	€ 228,162	€ 561,244	€ 15,870	€ 549,494	€ 27,620
Taxation									
Direct tax	€ 229,148	€ 100,172	€ 128,976	€ 140,898	€ 88,250	€ 224,631	€ 4517	€ 220,336	€ 8812
Indirect tax	€ 90,774	€ 38,939	€ 51,836	€ 54,887	€ 35,888	€ 88,278	€ 2496	€ 86,430	€ 4344
Gross tax	€ 319,922	€ 139,111	€ 180,812	€ 195,785	€ 124,138	€ 312,909	€ 7013	€ 306,766	€ 13,156
Outcomes									
Life years lived from age 40	36.67	15.12	21.55	24.44	12.23	28.81	7.87	24.44	12.23
Productive life years lived	20.15	6.60	13.55	9.77	10.39	17.89	2.26	17.34	2.82

**Fig. 4:**
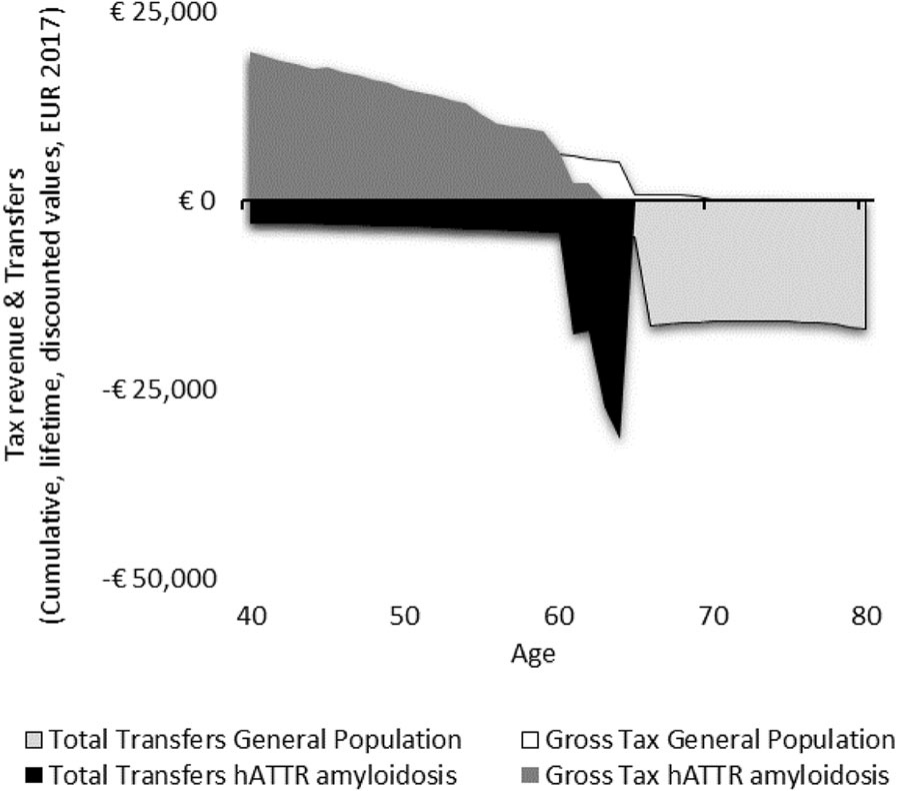
Lifetime transfers and taxes for Scenario 4

Scenario 4 estimates the fiscal course of an individual with hATTR amyloidosis with severe cardiomyopathy, where this main manifestation causes the patient to succumb due to early mortality within only 4 years, before reaching the latest stage of polyneuropathy. The health costs in this scenario are less significant than that of the other three scenarios (Fig. 4). This scenario estimates a reduction in lifetime taxes paid of €13,156 over a person in the general population.

## Discussion

All health conditions have the capacity to influence public economics beyond health service costs [1, 7]. The fiscal analysis described here illustrates how hATTR amyloidosis influences not only health costs, but also the cross-sectorial public economic burden attributed to lost tax revenues and public disability allowances to maintain living standards for people unable to work. Due to progressive disability, health care resource use, and early death, patients with hATTR amyloidosis with polyneuropathy receive more transfers from government compared to the general population. Disability and succumbing to early death decrease the labor force participation of a patient with hATTR amyloidosis, and thus their lifetime tax contribution compared to a person without hATTR amyloidosis in the general population from ages 40 to 80. When patients suffer from severe cardiomyopathy as part of hATTR amyloidosis, this main manifestation causes the patient to succumb to early mortality within only 4 years, far before reaching the latest stage of polyneuropathy, as such the health costs are less significant than that of the other three scenarios and even lower than expected in the general population. Due to the progressive nature of hATTR amyloidosis used in our case study, public costs including disability increase as the disease progresses with reduced lifetime tax contributions. The results indicate that halting disease progression early in the course of the disease would generate fiscal benefits beyond health benefits for patients. On the other hand, only slowing down the course of the disease would even strongly increase the total transfer costs as reflected in scenario 2 compared to scenario 1. The results described here in all scenarios illustrate how early mortality from hATTR amyloidosis can lead to pension cost savings for government. However, considering the costs of social protection that are paid to people with hATTR amyloidosis there is no overall saving to the public system. This study illustrates the cross-sectorial impact of health conditions that create dependency on social programs due to the inability to work. The results described here are likely an underestimate of the fiscal losses as many people with hATTR amyloidosis will rely heavily on a close relative that may have to quit their job or have to reduce work activity which further translates to reduced tax revenues for government.

In recent years with the widespread application of cost-effectiveness analysis to inform allocation decisions in healthcare, many have questioned whether this methodological approach is appropriate for rare diseases and orphan drugs [40, 41]. As previously mentioned here, all health conditions can have fiscal consequences, however there are some features of rare diseases which could result in greater fiscal impact compared to adult onset chronic conditions. For instance, many rare diseases impact from birth, are severely disabling and can greatly reduce life-expectancy [40], all features that can give rise to fiscal consequences in comparison to other chronic health conditions that often start later in life. With this in mind many, have noted the need to consider all elements of value and to ensure the full value of orphan drugs is reflected to paying audiences [40, 42]. In the framework described here, we reflect the government perspective which captures the fiscal transactions between citizens and state – none of which are captured by the existing cost-effectiveness perspectives of health service and societal. The government perspective is a meso-level perspective between health and society.

Unlike health costs, public costs are often defined by statutory disability payment rates, and various public allowances, therefore they are capped and are the same regardless of the underlying disease. Access to these benefits is defined by disability status and the age at which they are accessed. For example, a person claiming disability due to heart disease or any other condition would be entitled to the same public benefits as a person with hATTR amyloidosis as long as the disability level was deemed to be equivalent. Therefore, the fiscal results described here are applicable to other medical conditions that have a comparable impact on lifetime productivity as shown here for hATTR amyloidosis. Furthermore, as described here, governments lose money from health conditions based on earning capacity and ability to pay taxes. Similar estimates could be derived for other rare diseases applying this approach.

The main public economic variation between conditions is the age of disease onset and rate of progression which causes someone to become disabled. Diseases that impact early life can mean lower lifetime earnings, lower savings and lower wealth accumulation which can influence living standards in retirement. This would suggest that the ability to prevent health events or halt disease progression early in the disease course may enable people to remain productive: this will have benefits on earnings and wealth accumulation, as well as reducing demand for public benefits now and in the future. However, this does highlight one of the weaknesses of fiscal modeling that would tend to favor treating younger cohorts. Whilst this does pose equitable challenges, this is a fiscal reality and highlights the interdependence between generations in terms of taxes paid and benefits received. Despite these limitations, we believe this analysis highlights the need for a broader perspective when applied to health as the conventional cost-effectiveness framework used by many health technology assessment (HTA) agencies typically does not account for other costs beyond direct healthcare costs.

Excluding health related costs, the broader fiscal consequences of health conditions are consistent across diseases. Governments pay statutory amounts for disability regardless of the underlying health condition. Hence, the results shown here are representative of what any individual might receive who is disabled for any health condition such as cardiovascular disease, arthritis, renal failure or hATTR. In this regard the non-health related fiscal impacts described here are directly transferable to other health conditions in the Netherlands based on age-specific statistical averages. The variation in fiscal costs that is observed across rare diseases is mostly attributable to direct health costs. Furthermore, from our experience we observe consistency across many Western European democracies. Hence, we would expect a similar pattern of social spending and reductions in lifetime tax contributions to exist in other European markets [43].

Healthcare in the Netherlands is financed through a statutory health insurance scheme with regulated competition, with prevention and social insurance financed through taxation. The government is responsible for setting priorities and monitoring costs and quality through a range of agencies [44]. New pharmaceuticals are evaluated by the Zorginstituut Nederland in which a societal perspective is applied with emphasis on health costs. In contrast, the fiscal modeling approach described here is between the health sector and societal perspective reflecting specific financial transactions between citizen and state. The added value of the fiscal approach i.e. “government perspective”, is that tax revenue losses and gains can be accounted for based on investments in healthcare. In this regard, fiscal modeling helps to illustrate that health and healthcare are not only a cost, but also an investment, that can influence public accounts based on health spending.

Fiscal models in health can inform a range of government stakeholders in relation to how changes in morbidity and mortality influence public accounts. In the case of hATTR amyloidosis, the public economic burden is relatively small due to low prevalence, however applying a broader public health burden on this type of analysis can be more informative. For example, previous studies have explored the fiscal consequences of diabetes and obesity which can be in the billions of dollars [11]. A cross-sectorial public economic analysis as described here can inform a range of different government sectors regarding future cost and revenue implications.

Introducing fiscal models in healthcare decision making can introduce new factors to consider in resource allocation decisions. Firstly, the assessment indicates there are many more public costs than simply looking at the health costs. This is an important consideration as health systems in developed countries are mostly tax financed in the same manner as other pay-as-you-go public programs. Therefore, health investments that delay progression or prevent events from occurring offer fiscal benefits for government in terms of future taxes and delaying disability payments. Secondly, considering that many public sector costs attributed to rare diseases are often non-health related, we must gauge how these additional public cost elements should be considered in relation to pricing of new interventions that slow down progression and/or prevent morbidity and mortality. Perhaps most challenging is how to rationalize the observation that early mortality can save costs for government. There are no clear answers for this, however from our own work we have observed situations where conclusions from fiscal models can conflict those from cost-effectiveness models [45].

## Conclusions

The public economic framework described here can be an added approach for estimating disease burden and therapeutic interventions for rare diseases. We believe this approach can be applied to a range of different rare diseases and offers a complementary approach to cost-effectiveness analysis and can be used for priority setting.

## Supplementary information

**Additional file 1: Table S1.** Cost data for hATTR by PND stage in Euros over 6 months. **Table S2.** Cost data for hATTR patients who also have cardiomyopathy (by severe and non-severeness) in Euros over 6 months.

**Additional file 2: Table S3.** Cost per hATTR amyloidosis disease state based on best supportive care (BSC) per 6-month period in 2018 € (*).

## Data Availability

The results described here are a modeling study comprised from secondary data sources. No primary data collection was performed in relation to this work. All supporting data used for constructing the model is available in the public domain and has been cited or has been provided directly in the manuscript. The results from a survey that was performed to estimate health costs is provided in Additional file 2. Table S3.
