# Supplementary material for: Estimating the fiscal impact of rare diseases using a public economic framework: a case study applied to hereditary transthyretin-mediated (hATTR) amyloidosis
Source: Orphanet J Rare Dis. 2019 Sep 18;14:220. doi: 10.1186/s13023-019-1199-x (PMC6751602; doi:10.1186/s13023-019-1199-x)
Supplement: Supplementary file 1 — Additional file 1: Table S1. Cost data for hATTR by PND stage in Euros over 6 months. Table S2. Cost data for hATTR patients who also have cardiomyopathy (by severe and non-severeness) in Euros over 6 months. [file 13023_2019_1199_MOESM1_ESM.pdf]

Additional file 1

Table S1. Cost data for hATTR by PND stage in Euros over 6 months

**Resource**	**Unit cost (per mg of drug)**	**PND I (EUR/ 6 months)**	**PND I (EUR/ 6 months)**	**PND II (EUR/ 6 months)**	**PND IIIa (EUR/ 6 months)**	**PND IIIb (EUR/ 6 months)**	**PND IV (EUR/6 months)**	**Source**	**Assumptions**
		Mean	Mean	Mean	Mean	Mean	Mean		
Tramadol	0.00		2.69	3.20	3.20	3.20	9.71	https://www.medicijnkosten.nl/TRAMADOL 200MG	
Amitritptyline	0.00		3.07	2.74	3.29	3.29	5.01	https://www.medicijnkosten.nl/AMITRIPTYLINE 25MG	
Lyrica (Pregabalin)	0.00		3.08	4.75	5.66	5.11	6.39	https://www.medicijnkosten.nl/LYRICA 300MG	
Gabapentin	0.00		14.44	22.19	26.30	23.83	30.13	https://www.medicijnkosten.nl/GABAPENTINE 300MG	
Morphine	0.01		0.37	1.41	1.83	1.83	4.75	https://www.medicijnkosten.nl/MORFINE 10MG	
Iktorivil (Clonazepam)	0.05		1.10	3.49	3.49	1.92	11.14	https://www.medicijnkosten.nl/CLONAZEPAM 2MG	
Footcare	33.00		20.04	29.70	33.00	29.70	35.36		Assumption, same as orthotics (Hermans 2018)
Midodrine (2,5-10 mg 3 times daily)	0.31		78.35	213.95	339.00	399.26	421.86	https://www.medicijnkosten.nl/MIDODRINE 5MG	

Fludrocortisone (50-300 microgram daily)	42.56		59.27	139.91	219.57	240.95	247.10	https://www.medicijnkosten.nl/FLUDROCORTISON 31,25MCG	
Topical Lidocaine patches (700 mg 1-3 times daily)	0.00		0.00	0.00	0.00	0.00	0.00		Not reimbursed
Duloxetine (60-120 mg daily)	0.00		4.38	8.00	8.66	8.66	7.01	https://www.medicijnkosten.nl/DULOXETINE 60MG	
Doxepin (75-300 mg daily)	0.00		18.64	32.03	32.03	32.03	18.90	https://www.medicijnkosten.nl/DOXEPINE 50MG	
Capsaicin cream (0,5 gram 3-4 times daily)	0.05		357.95	1124.97	1738.59	1738.59	2761.29	https://www.medicijnkosten.nl/CAPSAICINE 0,075%	
Paracetamol (500 mg 4-6 times daily)	0.00		0.00	0.00	0.00	0.00	0.00		Not reimbursed
Zopiclone or diazepam - sleeping tablets (5 -15 mg per night)	0.01		0.37	0.68	1.56	1.80	2.02	https://www.medicijnkosten.nl/ZOPICLON 7,5MG	
Orthotics	33.00		0.00	272.25	354.75	358.88	363.00	Hermans 2018	
**Gastrointestinal disorders**									
Flagyl (Metronidazol)	0.00		0.00	2.41	1.61	1.61	0.00	https://www.medicijnkosten.nl/METRONIDAZOL 500MG	
Ciprofloxacin	0.00		0.04	0.64	0.43	0.43	0.38	https://www.medicijnkosten.nl/CIPROFLOXACINE 500MG	

Flucloxacillin	0.00		1.17	0.00	0.00	0.00	0.00	https://www.medicijnkosten.nl/FLUCLOXACILLINE 1000MG	
Ampicillin	0.00		1.63	0.00	0.00	0.00	0.00	https://www.onmeda.de/AMPICILLIN 1000MG	
Loperamide	0.05		16.07	54.06	56.98	63.92	81.57	https://www.medicijnkosten.nl/LOPERAMIDE 2MG	
Sodium picosulphate	0.00		0.00	0.00	0.00	0.00	0.00		Not reimbursed
Parenteral nutrition (every other day)	306.94		0.00	112.11	112.11	504.50	403.60	Puffelen 2018	Inflated to 2017
Dietician (visits)	27.00		15.62	21.60	27.00	29.70	91.80	Hermans 2017	Inflated to 2017
Colostomy (procedure)	347.55		0.00	0.35	1.74	2.17	2.09	van Wunnik 2012	Inflated to 2017
Metoclopramide (10 mg 1-3 times daily)	0.00		0.52	1.02	1.52	1.58	2.58	https://www.medicijnkosten.nl/METOCLOPRAMIDE 10MG	
Codeine (15-60 mg 3-4 times daily)	0.02		20.82	81.54	143.13	156.14	232.77	https://www.medicijnkosten.nl/CODEINE 15MG	
Lactulose (15 mg 1-2 times daily)	0.00		0.10	0.19	0.40	0.43	0.59	https://www.medicijnkosten.nl/LACTULOSE 670MG/ML	
Octreotide (12,5-25 microgram daily)	67.60		3.86	10.03	14.66	14.81	16.67	https://www.medicijnkosten.nl/OCTREOTIDE 0,05MG	
Domperidone (10 mg 1-3 times daily)	0.01		0.24	0.97	1.27	1.52	2.10	https://www.medicijnkosten.nl/DOMPERIDON 10MG	

Movicol (1-3 sachets daily)	0.12		2.19	3.51	4.60	5.96	7.28	https://www.medicijnkosten.nl/MACROGOL SACHET 13,8G	
Senna (15-30 mg daily)	0.00		0.10	0.22	0.35	0.57	0.68	https://www.medicijnkosten.nl/SENNA 185MG	
Ondansetron (16 mg daily)	0.02		0.48	2.41	3.11	3.18	4.49	https://www.medicijnkosten.nl/ONDANSETRON 8MG	
**Bladder dysfunction**									
Catheterisation (procedure)	36.89		1.84	0.81	1.48	3.23	36.89	Van Den Broek 2011	Inflated to 2017
Suprapubic catheterisation (procedure)			0.00	0.00	0.00	0.00	0.00		
Antibiotic usage for recurrent UTIs (100 mg 4 times daily)	0.00		2.22	20.89	32.02	33.24	31.91	https://www.medicijnkosten.nl/NITROFURANTOINE 50MG	
Antibiotic usage for prophylaxis to prevent UTIs (100 mg daily)	0.00		0.40	2.71	4.82	5.70	4.27	https://www.medicijnkosten.nl/NITROFURANTOINE 50MG	
Anticholinergics, such as Oxybutinin (2,5 mg 3 times daily - 5 mg 4 times dialy)	0.01		0.50	2.56	4.29	4.72	5.73	https://www.medicijnkosten.nl/OXYBUTYNINE 2,5MG	

Tolterodine (2 mg 2 times daily)	0.06		0.67	1.67	3.42	3.42	4.08	https://www.medicijnkosten.nl/TOLTEROD INE 2MG	
**Occular problems**									
Vitrectomy	1428.15		9.52	71.41	0.00	0.00	57.13	Brouwer 2010	Inflated to 2017
Physician, Eye Clinic (visits)	93.11		34.91	46.55	46.55	46.55	37.24	Kostenhandleiding	Inflated to 2017
Pilocarpine hydrochloride (used daily)	0.02		0.00	0.00	0.00	0.00	0.00	https://www.medicijnkosten.nl/PILOCARPI NE 10MG/ML	
**Other**									
Citalopram	0.00		0.55	0.37	0.44	0.55	1.53	https://www.medicijnkosten.nl/CITALOPR AM 20MG	
Sertraline	0.00		0.85	0.68	0.73	0.91	2.02	https://www.medicijnkosten.nl/SERTRALI NE 100MG	
EPO (4000 IU every other week)	29.36		0.00	0.00	0.00	11.74	1.17	https://www.medicijnkosten.nl/EPOETINE ALFA 0,5ML	
Knee surgery (procedure)	6072.07		242.88	151.80	0.00	0.00	0.00	Hermans 2017	
Social welfare officer (visits)	66.50		13.30	33.25	49.88	49.88	66.50	Kostenhandleiding	Inflated to 2017
Psychologist (visits)	65.48	23.39	23.39	17.19	33.83	33.83	50.20	Kostenhandleiding	Inflated to 2017
Dental care (visits)	21.00		14.70	13.13	14.00	14.00	14.70	https://www.tandarts.nl/dental care	Inflated to 2017
Physician, specialist (visits)	93.11	59.85	86.46	112.50	124.14	139.66	199.51	Kostenhandleiding	Inflated to 2017

Hospitalisation days (days)	487.01		556.59	243.51	487.01	243.51	2713.36	Kostenhandleiding	Inflated to 2017
Urologist visits	93.11		18.62	38.17	51.21	102.42	162.93	Kostenhandleiding	Inflated to 2017
Viagra (50 mg before sexual activity)	0.16		34.32	114.40	85.80	100.10	20.02	https://www.medicijnkosten.nl/SILDENAFIL 50MG	
Acupuncture	60.00		45.00	45.00	45.00	15.00	0.02	http://www.acupunctuur-rotterdam.com/consultation	
**Primary care**									
Physician (visit)	33.76	24.12	41.00	41.64	58.52	61.79	85.61	Kostenhandleiding	Inflated to 2017
Physiotherapist (visits)	33.76		10.13	22.96	39.84	37.14	9.62	Kostenhandleiding	Inflated to 2017
Occupational therapists (visits)	23.87		1.19	8.95	21.48	23.87	59.68	Dorresteijn 2016, Chapter 6	Inflated to 2017
Nurse (visits)	12.54	3.13	6.08	4.70	15.67	34.48	325.99	Bosmans 2014	Inflated to 2017
Assistant nurse for wound dressings (visits)	12.54		8.02	2.09	8.36	8.36	325.99	Bosmans 2014	Inflated to 2017
Rehabilitation (days)	470.64		258.85	0.00	156.88	549.08	12236.74	Kostenhandleiding	Inflated to 2017
**Homecare**									
Home service (hours)	23.53		0.00	0.00	1302.11	3039.57	60165.93	Kostenhandleiding	Inflated to 2017
Special housing (Days)	159.61		0.00	0.00	0.00	0.00	2234.54	Kostenhandleiding	Inflated to 2017
**One-off costs**									

Electric wheelchair	5381.00		0.00	0.00	336.31	874.41	1076.20	Kobelt 2006	
Manual wheelchair	358.00		0.00	0.00	85.03	120.83	0.00	Hermans 2017	
Stick	19.95		0.00	8.38	11.57	0.00	0.00	Hermans 2017	
Crutch	39.75		0.00	6.63	3.81	2.82	0.00	Hermans 2017	
Walking chair	109.00		0.00	0.00	13.63	13.63	81.75	https://www.vegro.nl/rollator move light	
Walking frame	59.99		0.00	0.75	5.85	43.64	9.75	https://www.vegro.nl/looprek vast verstelbaar	
Permobil	1139.00		0.00	0.00	142.38	256.28	740.35	https://www.vegro.nl/scootmobiel	
Shower chair	450.00		0.00	129.38	140.63	135.00	45.00	Hermans 2017	
Adjustment, kitchen	2694.00		0.00	673.50	583.70	89.80	0.00	Dorresteijn 2016, Chapter 6	
Adjustment, bathroom	2694.00		0.00	853.10	1661.30	179.60	0.00	Dorresteijn 2016, Chapter 6	
Door opener	18.87		0.00	0.94	6.13	0.00	0.00	Due to variety of door opener results, we assume that it is similar to the UK price. (1 GBP =1.12196EUR)	
Rails	37.95		0.00	6.33	22.77	2.53	0.00	https://www.efarma.nl/mobile rail 25cm	
Ramps	169.00		0.00	12.68	44.36	61.26	50.70	https://www.vegro.nl/rampkit 2	
Homecare bed including lift	2129.00		0.00	0.00	239.51	505.64	1383.85	Kobelt 2006	

Table S2. Cost data for hATTR patients who also have cardiomyopathy (by severe and non-severeness) in Euros over 6 months

**Resource**	**Unit cost (per mg of drug)**	**NTproBNP <3000 ng/ml**	**NTproBNP >3000 ng/ml**	Sources
		Mean	Mean	
Spironolactone (100-200 mg per day)	0.00 €	11.64 €	20.55 €	https://www.medicijnkosten.nl/SPIRONOLACTON 100MG
Eplerenone (25 mg per day)	0.01 €	6.30 €	12.60 €	https://www.medicijnkosten.nl/EPLERENON 25MG
Furosemida (20-40 mg per day)	0.00 €	5.41 €	7.12 €	https://www.medicijnkosten.nl/FUROSEMIDE 20MG
Bumetanide (1 mg per day)	0.03 €	2.10 €	3.10 €	https://www.medicijnkosten.nl/BUMETANIDE 1MG
Sando K (2-4 times daily)	0.00 €	0.07 €	0.07 €	https://www.medicijnkosten.nl/KALIUMCHLORIDE 75MG/ML
Bisoprolol (10-20 mg per day)	0.00 €	3.84 €	3.97 €	https://www.medicijnkosten.nl/BISOPROLOL 10MG
Amiodarone (200-600 mg per day)	0.00 €	17.53 €	19.72 €	https://www.medicijnkosten.nl/AMIODARON 200MG
Warfarin (3-9 mg per day)	0.78 €	255.09 €	255.09 €	https://www.medicijnkosten.nl/WARFARINE 5MG/ML
Rivaroxaban (20-100 mg per day)	0.11 €	401.48 €	464.21 €	https://www.medicijnkosten.nl/RIVAROXABAN 20MG
Dabigatran (110-220 mg per day)	0.01 €	54.31 €	50.13 €	https://www.medicijnkosten.nl/DABIGATRAN 110MG

Apixaban (5-10 mg per day)	0.22 €	98.54 €	119.44 €	https://www.medicijnkosten.nl/APIXABAN 5MG
Echocardiogram (procedure)	207.38 €	233.30 €	293.79 €	Ringborg 2008
Electrocardiogram (procedure)	22.52 €	30.02 €	35.65 €	Ringborg 2008
Cardiac MRI (procedure)	222.12 €	77.74 €	92.55 €	Miquel-Cases 2016
SA node ablation (procedure)	4,916.72 €	1,229.18 €	1,229.18 €	Ringborg 2008
iv diuresis (procedure)		- €	- €	
Hearth failure specialist nurse (visits)	12.54 €	12.54 €	28.84 €	Bosmans 2014
Cardiac rehabilitation (visits)	156.54 €	26.09 €	104.36 €	Kostenhandleiding
Cardiac technician (device checks and maintenance; visits)	12.54 €	10.66 €	11.28 €	Bosmans 2014
Accident and emergency (A&E) (visits)	264.99 €	119.25 €	304.74 €	Kostenhandleiding
Hospitalisations (days)	487.01 €	487.01 €	3,287.35 €	Kostenhandleiding

Percutaneous coronary intervention (PCI) (surgical procedure)	3,657.03 €	- €	- €	Ringborg 2008
Cardiac Resynchronization Therapy (CRT)	8,564.16 €	2,854.72 €	2,854.72 €	Geisler 2017
Permanent pacemaker	8,564.16 €	963.47 €	1,124.05 €	Geisler 2017
Intraventricular pacemaker	8,564.16 €	578.08 €	785.05 €	Geisler 2017
Implantable cardioverter defibrillator	#########	1,616.18 €	1,847.06 €	Ringborg 2008
Implantable loop recorder	890.89 €	75.73 €	14.85 €	Kanters, 2015
Holter monitors	125.61 €	17.35 €	21.35 €	Ringborg 2008
