# Supplementary material for: Estimating the fiscal impact of rare diseases using a public economic framework: a case study applied to hereditary transthyretin-mediated (hATTR) amyloidosis
Source: Orphanet J Rare Dis. 2019 Sep 18;14:220. doi: 10.1186/s13023-019-1199-x (PMC6751602; doi:10.1186/s13023-019-1199-x)
Supplement: Supplementary file 2 — Additional file 2: Table S3. Cost per hATTR amyloidosis disease state based on best supportive care (BSC) per 6-month period in 2018 € (*). [file 13023_2019_1199_MOESM2_ESM.pdf]

Table S3. Cost per hATTR amyloidosis disease state based on best supportive care (BSC) per 6-month period in 2018 € (*)

	Disease Stage	HCRU PN	HCRU CM	AEs	Total cost
NT proBNP< 3000 pg/mL	PND 0	1,10	9,188	355	9,653
	PND I	2,039	9,188	355	11,581
	PND II	3,147	9,188	355	12,690
	PND IIIA	5,727	9,188	355	15,270
	PND IIIB	8,198	9,188	355	17,741
	PND IV	83,624	9,188	355	93,166
NT proBNP≥3000 pg/mL	PND 0	110	12,991	355	13,457
	PND I	2,039	12,991	355	15,385
	PND II	3,147	12,991	355	16,493
	PND IIIA	5,727	12,991	355	19,073
	PND IIIB	8,198	12,991	355	21,544
	PND IV	83,624	12,991	355	96,970

(*) In this calculation the following costs are not considered: Health care resource use one off costs, end of life care, and orthotic liver transplant
